# Dermatophytes Activate Skin Keratinocytes via Mitogen-Activated Protein Kinase Signaling and Induce Immune Responses

**DOI:** 10.1128/IAI.02776-14

**Published:** 2015-03-17

**Authors:** Rebecca R. Achterman, David L. Moyes, Selvam Thavaraj, Adam R. Smith, Kris M. Blair, Theodore C. White, Julian R. Naglik

**Affiliations:** a Seattle Biomedical Research Institute, Seattle, Washington, USA; b Mucosal and Salivary Biology Division, King's College London Dental Institute, King's College London, London, United Kingdom; c Division of Cell Biology and Biochemistry, University of Missouri at Kansas City, Kansas City, Missouri, USA

## Abstract

Dermatophytes cause superficial and cutaneous fungal infections in immunocompetent hosts and invasive disease in immunocompromised hosts. However, the host mechanisms that regulate innate immune responses against these fungi are largely unknown. Here, we utilized commercially available epidermal tissues and primary keratinocytes to assess (i) damage induction by anthropophilic, geophilic, and zoophilic dermatophyte strains and (ii) the keratinocyte signaling pathways, transcription factors, and proinflammatory responses induced by a representative dermatophyte, Trichophyton equinum. Initially, five dermatophyte species were tested for their ability to invade, cause tissue damage, and induce cytokines, with Microsporum gypseum inducing the greatest level of damage and cytokine release. Using T. equinum as a representative dermatophyte, we found that the mitogen-activated protein kinase (MAPK) pathways were predominantly affected, with increased levels of phospho-p38 and phospho-Jun N-terminal protein kinase (JNK) but decreased levels of phospho-extracellular signal-regulated kinases 1 and 2 (ERK1/2). Notably, the NF-κB and PI3K pathways were largely unaffected. T. equinum also significantly increased expression of the AP-1-associated transcription factor, c-Fos, and the MAPK regulatory phosphatase, MKP1. Importantly, the ability of T. equinum to invade, cause tissue damage, activate signaling and transcription factors, and induce proinflammatory responses correlated with germination, indicating that germination may be important for dermatophyte virulence and host immune activation.

## INTRODUCTION

Dermatophytes are pathogenic fungi that can infect skin, hair, and nails. They are common superficial fungal infections, estimated to affect more than 20% of the world's population (1). In addition to causing cutaneous mycoses in immunocompetent hosts, dermatophytes are also associated with invasive disease in immunocompromised hosts (2, 3). Despite the high prevalence of dermatophyte infections, our understanding of the pathogenic mechanisms of these organisms is lacking (4, 5). Dermatophyte species differ in the infections they cause (6). Anthropophilic (human-adapted) species tend to cause chronic infections with minimal inflammation, while infections by zoophilic (animal-associated) or geophilic (soil-dwelling) species tend to cause an acute, inflammatory disease. The recent sequencing of several dermatophyte genomes will help develop hypotheses about which gene products play a role in virulence and niche adaptation (7–9).

While dermatophytes (e.g., Arthroderma benhamiae) are known to invade human epidermal tissues (10), the host response to dermatophyte infection is incompletely understood. Epidermal keratinocytes secrete cytokines in response to dermatophytes (11, 12), but the mechanisms that regulate these host response are largely unknown (13). Here, we utilized commercially available epidermal tissues and primary keratinocytes, as an alternative to animal models, to assess dermatophyte virulence and the host response. Infections were performed with anthropophilic (Trichophyton rubrum, Trichophyton tonsurans), geophilic (Microsporum gypseum), and zoophilic (Trichophyton equinum, Microsporum canis) dermatophytes, using the five dermatophyte strains whose genome sequences were recently released with annotation (8). The abilities of these dermatophytes to invade, cause tissue damage, and induce proinflammatory cytokines were compared. Furthermore, we determined whether different morphological forms of the dermatophyte Trichophyton equinum differentially activated cell signaling pathways and host transcription factors.

## MATERIALS AND METHODS

### Fungal strains and growth conditions.

The following dermatophyte strains have been characterized elsewhere (5, 8, 14): Trichophyton rubrum (CBS118892), Trichophyton tonsurans (CBS112818), Trichophyton equinum (CBS127.97), Microsporum canis (CBS113480), and Microsporum gypseum (CBS118893). Dermatophytes were grown on YEPD (10% yeast extract, 20% peptone, 2% dextrose) at 30°C to obtain mycelial growth. Agar (BactoAgar; Difco) was added at 20 g per liter where required. Conidia were isolated after 10 days of growth on MAT agar (10% solution of Sabauroud medium with 0.1% [final volume] MgSO_4_-7H_2_O and KH_2_PO_4_ and agar added at 20 g per liter) at 30°C as described previously (14). Briefly, the surface of the plate was scraped with 20% Tween 20 to collect conidia and mycelia, and then this suspension was filtered through a layer of Miracloth (Calbiochem) to remove mycelia. The conidia were centrifuged (3,000 × *g*, 5 min), washed twice in phosphate-buffered saline (PBS), and resuspended in 1 ml PBS (Gibco). The conidial concentration was determined by using a hemocytometer, and CFU/μl was determined using plate counts on YEPD after 72 h of growth at 30°C. For germination before being added to human cells, conidia were diluted in liquid YEPD medium at a concentration of 2 × 10^8^ CFU/ml and incubated at 30°C overnight in a slow-shaking incubator (60 to 80 rpm). Under these conditions, over half the conidia germinated, but minimal hyphal branching was observed. The solution containing conidia and germinated conidia was washed and resuspended in PBS (Gibco) before inoculations (see “Infection of commercial EpiDerm tissues” below). While this represents a mixture of germinated and nongerminated conidia, 100% germination of conidia for dermatophytes is difficult to achieve under many conditions. Using these germinated conidia mimics hyphae as an inoculum, where quantification of hyphae in a mycelial mat is not possible.

### Primary keratinocytes.

Primary human keratinocytes were obtained from healthy donors (*n =* 3) and were a kind gift from Frank Nestle (King's College, London, United Kingdom). Normal human skin, from discarded plastic surgery specimens, isolated from patients attending dermatology clinics, were obtained in accordance with the Helsinki Declaration and approved by the Institutional Review Board of Guy's and St. Thomas' NHS Foundation Trust Hospital (06/Q0704/18). Written informed consent was obtained from all patients and healthy skin donors. Skin samples were cut into strips and incubated in Dispase II at 5 U/ml (Roche) in Hanks' balanced saline solution (HBSS; Gibco) at 4°C for 18 h. Epidermis was separated from dermis and incubated at 37°C for 15 min in trypsin-EDTA solution (Gibco). The keratinocyte suspension was filtered, and cells were washed twice at 500 × *g* for 5 min prior to resuspension in serum-free keratinocyte basal medium (KBM) supplemented with KGM Gold singlequots (Lonza). Cells were maintained at 37°C in a humidified atmosphere containing 5% CO_2_ and used in passages 3 to 6. Prior to the assay, cells were incubated in supplement-free KBM for 2 h.

### Infection of primary keratinocytes.

Primary keratinocytes were infected with either T. equinum conidia or germinated conidia at a multiplicity of infection (MOI) of 10 (5 × 10^6^ conidia added to 5 × 10^5^ keratinocytes) in supplement-free KBM. After incubation, culture supernatants were collected for lactate dehydrogenase (LDH) and cytokine assays. Cells were lysed with 120 μl of modified RIPA buffer (15) with protease (Pierce, United Kingdom) and phosphatase (Sigma, United Kingdom) inhibitors. All experiments were performed with keratinocytes from three different donors.

### Infection of commercial EpiDerm tissues.

Commercial epidermal tissues comprising normal human keratinocytes (EpiDerm from MatTek, catalog number EPI-200-AFAB) were cultured in New Maintenance culture medium without antifungals (NMM from MatTek, catalog number EPI-100-NMM-AFAB). Immediately upon arrival, inserts containing epidermal tissues were transferred to 6-well plates containing 5 ml prewarmed NMM and two metal washers onto which the inserts were placed. For tissue damage and cytokine detection assays, the tissues were allowed to equilibrate for 1 h at 37°C in 5% CO_2_ before infections were performed in triplicate. Conidia from different dermatophytes were added to tissues in 10 μl of PBS (10^4^ to 10^7^ conidia per tissue). Dermatophyte mycelial plugs were obtained by using the thin end of a Pasteur pipette to transfer a fungal plug from a YEPD plate. For the pilot experiment, two sizes of mycelial plugs were compared: large (the large opening of a sterile P200 tip) and small (the thin end of a Pasteur pipette). Future experiments used the thin end of a Pasteur pipette (“small” size), since this was found to induce damage and was easier to transfer than the large size. After inoculation, tissues were incubated at 30°C in 5% CO_2_. NMM was removed and replaced with 5 ml prewarmed NMM every 48 h. Spent medium was stored for further analysis at 4°C (tissue damage) or −80°C (cytokine detection). For histology and quantitative PCR, EpiDerm tissues were prepared as described above but were first allowed to equilibrate overnight at 30°C at 5% CO_2_ prior to infection with T. equinum conidia or germinated conidia (10^7^ CFU) in 100 μl of PBS. Prior to infection with germinated conidia, the percent germination was determined microscopically and used to calculate the required volume of ungerminated conidia for the conidial infections. Therefore, for each set of infections, the number of conidia matched the number of germinated conidia. For histology, tissues were removed from the membrane after 96 h of infection and stored in 10% neutral buffered formalin (Sigma; HT5012) at room temperature prior to analysis. For RNA analysis by quantitative PCR, tissues were isolated after 6 h and 24 h of infection, rinsed in PBS, and then lysed using 300 μl RNA lysis buffer (Sigma; L8285 with added beta-mercaptoethanol). The solution was incubated for 3 min on ice and then clarified in a refrigerated microcentrifuge for 10 min and stored at −80°C.

### Tissue damage assay.

Detection of LDH was used as a measure of tissue damage. LDH release from EpiDerm models was detected using an *in vitro* toxicology assay kit (Sigma; catalog number TOX-7) by following the manufacturer's protocol. Spent culture medium from three biological samples was removed for LDH measurement every 2 days, when the medium was changed.

LDH release from primary keratinocytes was detected using the CytoTox 96 nonradioactive cytotoxicity assay kit (Promega, United Kingdom) according to the manufacturer's protocol.

### Cytokine detection.

To detect cytokines secreted by epidermal tissues during dermatophyte infections, the BD cytometric bead array was used. The human inflammatory cytokines kit (BD Biosciences; catalog number 551511) contains phycoerythrin (PE)-conjugated anti-human interleukin 1β (IL-1β), IL-6, IL-8, IL-10, tumor necrosis factor alpha (TNF-α), and IL-12p70 antibodies. Antibodies recognizing human granulocyte colony-stimulating factor (G-CSF) and granulocyte-macrophage colony-stimulating factor (GM-CSF) were purchased as a flex set (BD Biosciences; catalog numbers 558335 and 558326). A total of 50 μl of spent medium (day 2, 4, 6, or 8 postinfection) or standard dilution was used in the culture supernatant assay procedure as described in the manufacturer's protocol. Detection was performed on a BD LSR II flow cytometer (BD Biosciences) and analyzed with FCAP array software (Soft Flow Hungary Ltd. for BD Biosciences). To detect cytokines secreted by primary keratinocytes, cell culture supernatant was collected 24 h postinfection. A total of 25 μl of the cell culture supernatant was used to determine cytokine levels for IL-1α, IL-1β, IL-6, IL-8, G-CSF, and GM-CSF by using the Fluorokine MAP cytokine multiplex kits (R&D Systems, United Kingdom), using a modified manufacturer's protocol. The panel of cytokines chosen for this assay was based on cytokines that have been shown to be produced in epithelial cells (15–20). Briefly, antibody-linked microbeads were incubated with neat cell culture supernatants in 25 μl followed by biotinylated detection antibody in 25 μl. The beads were read using a Bio-Plex 200 microbead flow cytometer. The data were analyzed using the Bio-Plex Manager 5.0 software suite (Bio-Rad, United Kingdom) using Logistic 5PL analysis.

### RNA extraction and analysis.

RNA was isolated using the GenElute total RNA extraction kit (Sigma-Aldrich, United Kingdom) and treated with a Turbo DNA-*free* kit (Life Technologies, United Kingdom) to remove genomic DNA. cDNA was generated using HIV reverse transcription (RT) (Life Technologies, United Kingdom), and then real-time PCR was performed using Jumpstart SYBR green mastermix (Sigma-Aldrich, United Kingdom) on a Rotor-Gene 6000 (Qiagen) by using the following program: 98°C for 3 min followed by 40 cycles of 98°C for 3 s, 60°C for 10 s, and 72°C for 20 s. Primers were taken from PrimerBank (http://pga.mgh.harvard.edu/primerbank/) (21) and are listed in Table 1.

**TABLE 1:** Primers used for quantitative PCR analysis of proinflammatory genes

Gene	Sense primer	Antisense primer
*mmp1*	5′-ACTCTGGAGTAATGTCACACCT-3′	5′-GTTGGTCCACCTTTCATCTTCA-3′
*timp1*	5′-GGGTTCCAAGCCTTAGGGG-3′	5′-TTCCAGCAATGAGAAACTCCTC-3′
*cox2*	5′-ATATGTTCTCCTGCCTACTGGAA-3′	5′-GCCCTTCACGTTATTGCAGATG-3′
*mkp1*	5′-GGCCCCGAGAACAGACAAA-3′	5′-GTGCCCACTTCCATGACCAT-3′
c-*fos*	5′-GGGCAAGGTGGAACAGTTATC-3′	5′-CCGCTTGGAGTGTATCAGTCA-3′

### Immunohistochemistry of EpiDerm tissues.

Formalin-fixed epidermal tissues were embedded and processed in paraffin wax using a standard protocol. For each sample, 5-μm sections were prepared using a Leica RM2055 microtome and silane-coated slides. After sections were dewaxed in xylene, protein expression was determined using rabbit anti-human polyclonal antibodies for MKP1 (Santa Cruz Biotechnology) and c-Fos (Source Bioscience, United Kingdom) (1:10 and 1:100, respectively), and sections were counterstained with peroxidase-conjugated goat anti-rabbit secondary IgG antibody, followed by diaminobenzidine (DAB) chromogen detection as per the manufacturer's protocol. To visualize fungal cells, sections were stained using periodic acid-Schiff (PAS), counterstained with hematoxylin, and examined by light microscopy.

### Detection of signal protein activation in primary skin keratinocytes.

Phospho-p38, phospho-Jun N-terminal protein kinase (JNK), phospho-extracellular signal-regulated kinases 1 and 2 (ERK1/2), phospho-IκBα, and phospho-Akt were assayed for using the Bio-Plex Pro magnetic phosphoprotein detection assay (Bio-Rad, United Kingdom). Cell lysates were assayed at 200 μg protein/ml according to the manufacturer's protocol. Levels were normalized to the β-actin housekeeping protein and expressed as fold change relative to the uninfected control.

### Statistics.

Data were analyzed using either the Student *t* test or one-way analysis of variance (ANOVA). A *P* value of 0.05 or less was taken as being significant.

## RESULTS

### Dermatophyte dose optimization, growth, and damage of EpiDerm models.

**FIG 1:**
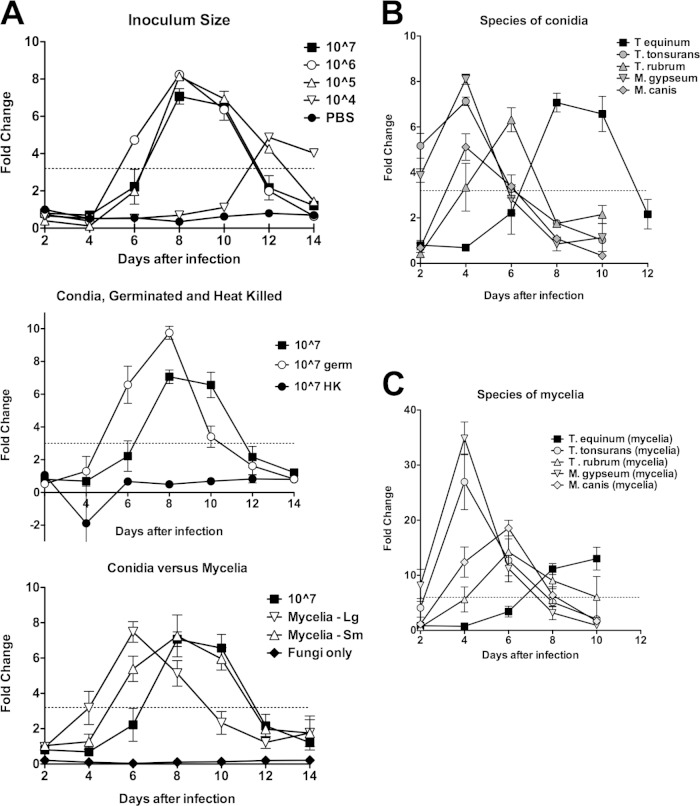
Dermatophytes cause LDH release by EpiDerm tissues. (A) Pilot experiment in which EpiDerm tissues were exposed to dermatophyte cells and LDH release was measured. Data from all graphs were obtained simultaneously. Values are expressed as fold change equal to the value divided by the day 2 PBS control average. Error bars represent standard errors. Top, inoculum size. A pilot experiment in which increasing amounts of T. equinum conidia were incubated with EpiDerm tissues. Experiments using 10^7^ conidia/tissue were performed in triplicate. Because of the limited number of EpiDerm samples in the experiment, the other levels of conidia were tested in a single biological sample. For 10^7^, values above the dotted line (3.2-fold change) were all statistically different (*P* < 0.05) from the PBS control. Statistics could not be performed on the other conidial concentrations. The PBS controls for all 14 days are shown. While they are not statistically significant, the values trend upward, suggesting that LDH is being released in PBS alone. Middle, germinated and heat-killed conidia. Samples were from the same experiment as shown in the top graph. The line for 10^7^ conidia is the same as that in the top graph, for comparison with germinated conidia and heat-killed conidia (see Materials and Methods). All samples were performed in biological triplicates. All values above the dotted line (3.2-fold change) are statistically significant (*P* < 0.05). Heat-killed conidia are not statistically different from PBS alone. Bottom graph, mycelial plugs. Large and small mycelial plugs (see Materials and Methods) were compared to the 10^7^ conidia and to a control with fungi but without tissues. All samples were performed in biological triplicates. All values above the dotted line (3.2-fold change) are statistically significant (*P* < 0.05). The “fungi alone” data are statistically lower than PBS data at all time points, demonstrating that fungi do not produce LDH on their own. (B) Tissues were infected with 10^6^ conidia from different dermatophyte species representing anthropophilic (T. rubrum, T. tonsurans), zoophilic (T. equinum, M. canis), or geophilic (M. gypseum) infections. Data points represent the average fold change from three biological triplicates, divided by the day 2 PBS control average. Error bars represent standard errors. Values above the dotted line (3.2-fold change) were all statistically different (*P* < 0.05) from the PBS control (except T. rubrum at day 4). Values below the dotted line were not statistically different (*P* < 0.05) from the PBS control (except at day 8 for T. tonsurans and T. rubrum). (C) Tissues were infected with mycelium plugs from the same species as in panel B. Mycelium plugs were obtained by using the thin end of a Pasteur pipette to transfer a fungal plug from a YEPD plate, and infections were performed in triplicate. LDH was detected in spent medium. Data points represent the average fold change from three biological triplicates, divided by the day 2 PBS control average. Error bars represent standard errors. Values above the dotted line (6-fold change) were all statistically different (*P* < 0.05) from the PBS control (except M. gypseum at day 2). Values below the dotted line were not statistically different (*P* < 0.05) from the PBS control (except at days 8 and 10 for T. tonsurans).

Pilot experiments were first undertaken with T. equinum to optimize infection conditions and to determine whether dermatophytes could grow and damage EpiDerm tissues. T. equinum was selected because it produces abundant conidia and causes an intermediate infection in humans (5, 14). Tissues were inoculated with 10^7^, 10^6^, 10^5^, or 10^4^ conidia/well (Fig. 1A, top), 10^7^ germinated conidia/well, 10^7^ heat-killed conidia/well (Fig. 1A, middle), and mycelial plugs/well (Fig. 1A, bottom). Negative controls included PBS (Fig. 1A, top) and fungi at 10^7^ viable conidia/well without tissue (Fig. 1A, bottom). Fungal growth was observed during a 14-day time course for all epidermal tissues inoculated with conidia, germinated conidia, or mycelial plugs, but no fungal growth was observed on tissues inoculated with heat-killed conidia or PBS.

Cytoplasmic LDH is released into the culture medium when cells are damaged or undergo necrosis and provides an indication of both the health of human EpiDerm tissues and progression of infection. As expected, cell damage was induced only in epidermal tissues inoculated with viable conidia, germinated conidia, or mycelial plugs (Fig. 1A). Damage induction was detected by day 6, when epidermal tissues were inoculated with 10^7^, 10^6^, and 10^5^ T. equinum conidia, which increased by day 8 but then gradually diminished as time progressed. At 10^4^ conidia, damage induction was not observed until day 12 (Fig. 1A, top). Therefore, an inoculum of 10^6^ conidia was chosen to compare the ability of five different dermatophytes (T. equinum, T. rubrum, T. tonsurans, M. canis, and M. gypseum) to damage EpiDerm models (Fig. 1B). In all cases, fungal growth was visible to the naked eye. T. tonsurans and M. gypseum induced damage by day 2, and the remaining dermatophytes induced damage by days 4 to 6 (Fig. 1B). These data demonstrate that dermatophytes are able to grow, invade, and cause tissue damage in the EpiDerm model of skin.

### Different dermatophyte species cause variable cytokine release.

**FIG 2:**
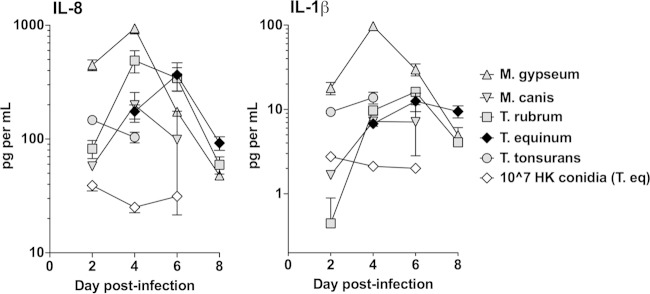
Dermatophyte infection results in cytokine release by EpiDerm tissues. Tissues were infected with 10^6^ conidia from the five dermatophyte species, as described for Fig. 1. The control is 10^6^ heat-killed conidia of T. equinum. Time points were chosen for each species that encompassed the peak LDH release determined in Fig. 1. Cytokines IL-8 and IL-1β were detected using the cytometric bead array kit. Data points represent the averages from three biological triplicates ± standard errors of the mean (SEM). For IL-8, the results with all live species conidia were statistically different (*P* < 0.05) from heat-killed conidia at days 2 and 4 (and all species except M. canis at 6 days). The results for M. gypseum conidia were significantly higher (*P* < 0.05) than those for all other species on days 2 and 4. For IL-1β, the results with all live species conidia were statistically different (*P* < 0.05) from heat-killed conidia on days 2 and 4 (and all species but M. canis at 6 days). The results for M. gypseum conidia were significantly higher (*P* < 0.05) than those for all other species on days 2 and 4.

The production of inflammatory cytokines and chemokines from EpiDerm models was assessed during the damage induction period (days 2 to 8) by all five dermatophytes. IL-8 and IL-1β secretion was elevated in tissues inoculated with all dermatophytes compared with either the PBS control or heat-killed (T. equinum) conidia (*P* < 0.05, Student *t* test) (Fig. 2). Interestingly, infection with M. gypseum induced statistically higher levels of these two cytokines than the other four dermatophytes (Fig. 2). IL-6, IL-10, TNF-α, IL-12p70, G-CSF, and GM-CSF were not induced from EpiDerm models (data not shown).

Dermatophytes can lose their ability to conidiate after repeated passage in the laboratory. Therefore, to confirm that the characteristics of mycelial and conidial infection were similar, mycelial plugs of the same five species were used to infect EpiDerm models. Highly similar profiles of cell damage were observed for all dermatophyte mycelial plug infections (Fig. 1C) when compared with conidial infections (Fig. 1B). Furthermore, selected analysis of T. rubrum and M. gypseum mycelial plug infections demonstrated IL-8 and IL-1β production, with both cytokines being secreted in larger amounts following M. gypseum infection (data not shown). IL-6, IL-10, TNF-α, and IL-12p70 were not detected (data not shown).

### Comparison between EpiDerm models and primary keratinocytes.

**FIG 3:**
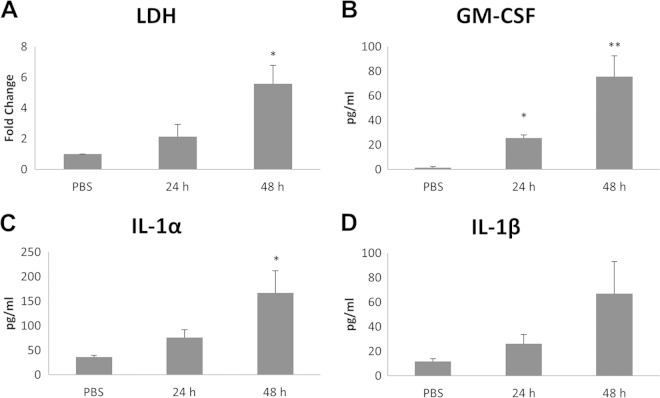
Dermatophyte infection of primary keratinocytes causes damage and cytokine release. T. equinum infections were performed for three separate donor isolations of keratinocytes with 10^6^ conidia for 24 and 48 h. (A) Detection of LDH in spent medium; (B to D) detection of cytokines by Luminex microbead assay in spent medium at 24 and 48 h postinfection. Data points represent the averages from three biological triplicates with separate donor primary keratinocytes ± SEM. Statistics were performed comparing postinfection cells with uninfected, 0-h-time-point cells. *, *P* < 0.05; **, *P* < 0.01.

Previous work has demonstrated that while organotypic tissue models and monolayer cells show similar responses to the fungal pathogen Candida albicans, monolayer cells tend to be more susceptible to damage induction and secrete a slightly different cytokine profile (15, 22, 23). Therefore, we assessed damage induction and cytokine release in primary keratinocytes isolated from healthy volunteers (*n =* 3) after infection with T. equinum conidia and compared the data with the EpiDerm models. As expected, a similar pattern of damage induction was observed between the two model systems, with a significant increase in LDH release 48 h postinfection in primary keratinocyte monolayers (5.5-fold increase [*P* < 0.05]) (Fig. 3A). Furthermore, T. equinum induced significant increases in IL-1α, IL-1β, and GM-CSF secretion by 48 h (Fig. 3B to D), which correlated with induction of cell damage, but IL-6 and G-CSF were not detected. This confirms that primary keratinocytes and organotypic EpiDerm models are similarly damaged by dermatophytes but slight differences exist in the secreted cytokine profile.

### Signal pathway activation by T. equinum.

**FIG 4:**
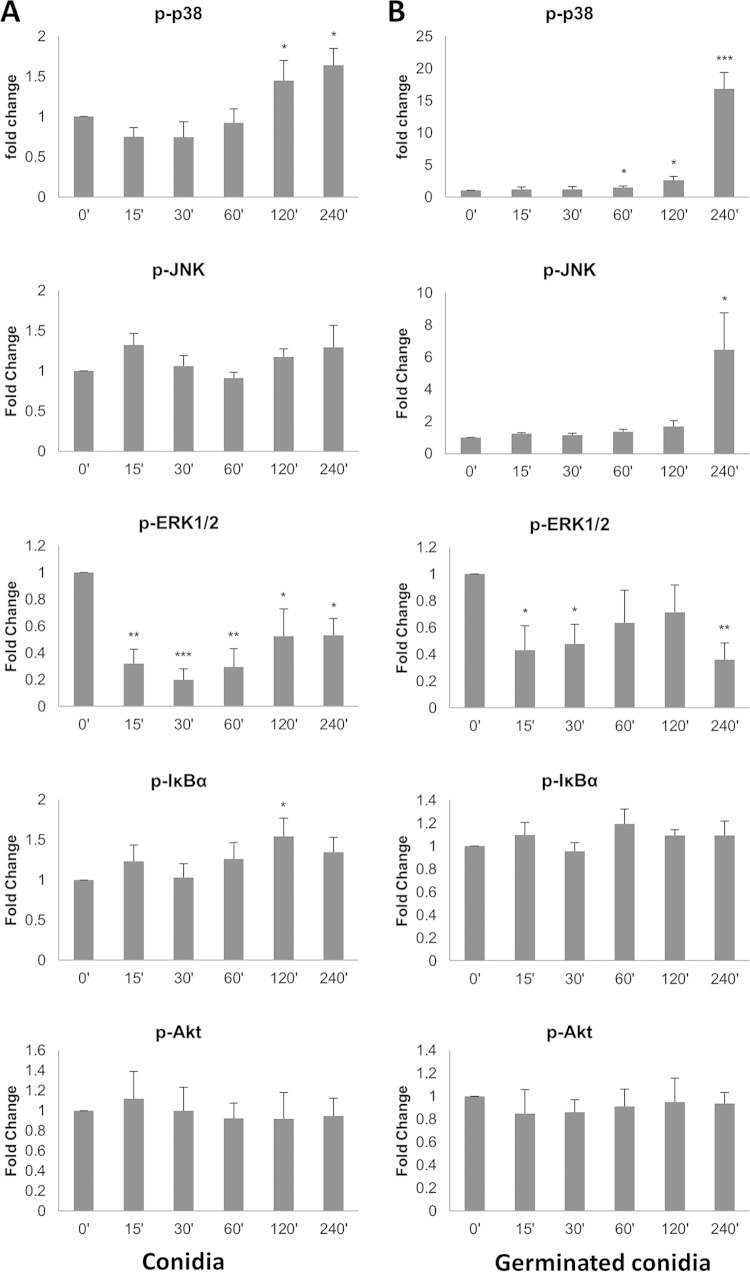
Dermatophyte infection of primary keratinocytes activates MAPK signal pathways. T. equinum infections were performed for three separate donor isolations of keratinocytes with 10^6^ conidia (A) or pregerminated conidia (B). Data points represent the averages from three biological triplicates with separate donor primary keratinocytes ± SEM. Statistics were performed comparing postinfection cells with uninfected, 0-h-time-point cells. *, *P* < 0.05; **, *P* < 0.01; ***, *P* < 0.001.

Given that T. equinum induced cytokine production in a measured response in both EpiDerm models and primary keratinocytes, we used T. equinum as a representative dermatophyte to identify the signaling pathways activated by these fungi. Primary keratinocyte monolayers need to be used for signaling experiments, as this allows uniform activation of cells, unlike the EpiDerm models, which are comprised of multilayered keratinocytes, thereby preventing uniform cell activation. Three signaling pathways were targeted, as these have been shown to be activated by other fungal pathogens (20, 24–28): mitogen-activated protein kinase (MAPK) comprising p38, ERK, and JNK; nuclear factor kappa-light-chain-enhancer of activated B cells (NF-κB); and phosphoinositol-3-kinase (PI3K). Further, to identify any differences in the signal pathways activated by the different morphological forms of dermatophytes, primary keratinocytes were infected with either conidia (Fig. 4A) or germinated conidia (Fig. 4B) for up to 4 h. Infection with conidia resulted in a low but significant increase (1.5-fold, *P* < 0.05) in phospho-p38 at 2 to 4 h postinfection, whereas infection with germinated conidia resulted in stronger increases in phospho-p38 with a 1.5-fold increase 1 h postinfection (*P* < 0.05), rising to an ∼17-fold increase 4 h postinfection (*P* < 0.01). Levels of phospho-JNK were unchanged throughout the 4-h time course with conidial infection but rapidly increased with germinated conidia, with a 6.5-fold increase 4 h postinfection (*P* < 0.05). In contrast, infection with either conidia or germinated conidia rapidly decreased phospho-ERK levels to 30 to 40% (*P* < 0.01; *P* < 0.05) of resting levels 15 min postinfection, which remained low throughout the time course. Although statistically the levels of phospho-IκBα increased at 2 h with conidia, there was no clear trend in activation for either conidia or germinated conidia. Phospho-Akt levels remained unchanged at all time points.

### Expression of proinflammatory genes and proteins.

**FIG 5:**
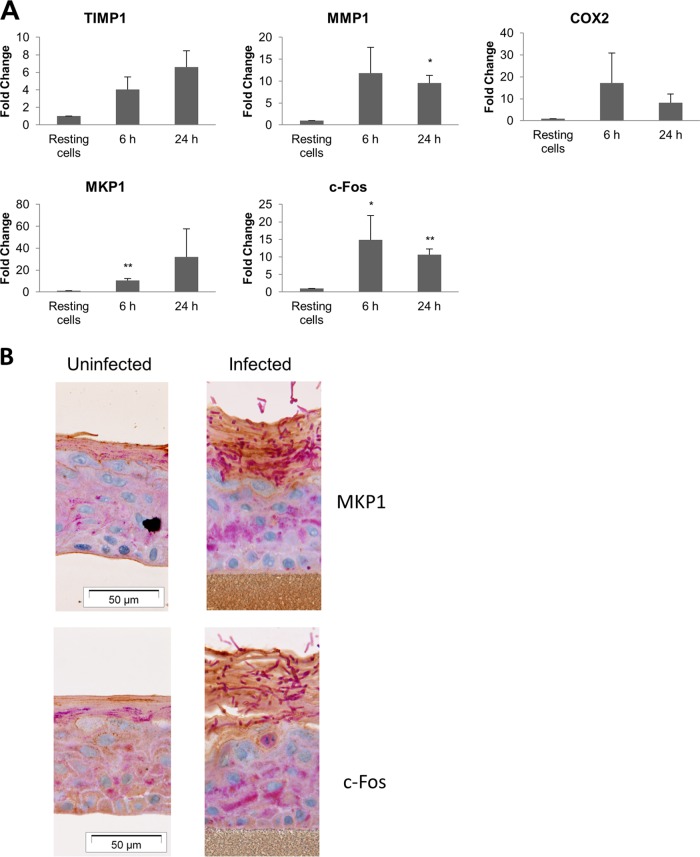
Dermatophyte infection of EpiDerm tissues results in expression of tissue remodeling and proinflammatory genes and MAPK signaling proteins. T. equinum infections were performed in triplicate with germinated or ungerminated conidia. (A) Expression of tissue remodeling (*timp1* and *mmp1*), proinflammatory (*cox2*), signal pathway regulatory (*mkp1*), and transcription factor (*cfos*) genes after infection. Data points represent the averages from three biological triplicates ± SEM. Statistics were performed comparing postinfection cells with uninfected, 0-h-time-point cells. *, *P* < 0.05; **, *P* < 0.01. (B) Expression of the MAPK pathway-associated regulator, MKP1, and the MAPK transcription factor, c-Fos, in infected EpiDerm tissue 96 h postinfection.

In addition to activating signaling pathways and inducing cytokine production, we also determined whether T. equinum infection of epidermal tissue upregulated other human genes associated with inflammation, including the tissue remodeling matrix metalloprotease *mmp1*, its regulatory gene *timp1*, and the proinflammatory mediator gene *cox2*. These analyses were performed with germinated conidia at 6 h and 24 h since, like signal pathway activation, gene induction is an early event and corresponds to the initial epidermal response to dermatophyte infection. Germinated conidia induced greater expression of all three genes by 6 h (Fig. 5A), which was generally maintained after 24 h. Other important regulators of epithelial inflammation are the transcription factor, c-Fos, and the MAPK phosphatase, MKP1, which we previously identified as playing an important function in recognizing pathogenic (hyphal) forms of C. albicans (15, 16). Here, we demonstrate that germinated T. equinum conidia significantly increased both c-Fos and MKP1 expression after 6 h and 24 h. Expression of c-Fos and MKP1 protein (Fig. 5B; dark brown stain in the surface layers) was confirmed by immunohistochemical analysis of the epidermal models at later time points (after 96 h to enable germination and penetration), with expression correlating with dermatophyte invasion (deep purple filaments at the surface).

## DISCUSSION

Dermatophytes are prolific pathogens and cause some of the most well-known cutaneous infections in humans (7, 29). However, little is known about how dermatophytes activate signal transduction pathways in host tissues, leading to an immune response. Utilizing a combination of organotypic epidermal models and primary keratinocytes, we identified MAPK signaling as a key pathway activated by dermatophytes, leading to the expression of the c-Fos transcription factor and cytokine induction.

To assess fungal virulence, we first determined the damage and cytokine-inducing ability of five different dermatophytes using organotypic EpiDerm models. All five were capable of inducing damage and cytokines, albeit to various degrees, confirming the utility of the EpiDerm tissues as surrogate models for the analysis of human dermatophyte skin infections. This supports other studies showing damage induction by other dermatophytes (A. benhamiae) by using human epidermal tissues comprising carcinoma keratinocytes (10). In our study, infection with the M. gypseum geophile generally induced higher levels of damage than the anthropophiles (T. rubrum, T. tonsurans) or zoophiles (T. equinum, M. canis), consistent with its role as a more virulent dermatophyte (14). This might also explain why certain cytokines, notably IL-1β, were detected at significantly higher levels from epidermal tissues inoculated with M. gypseum than from those inoculated with most other dermatophytes and the uninfected control. Although IL-8 production from primary keratinocytes infected with T. mentagrophytes, T. tonsurans, and T. rubrum (12) and IL-1β production from carcinoma keratinocytes infected with A. benhamiae (11) have previously been reported, our study is the first to demonstrate IL-1β production from primary human keratinocytes by T. equinum. Since IL-1β is induced via inflammasomes in immune cells (30), our data strongly suggest that dermatophytes activate keratinocyte inflammasomes during infection. Recently, M. canis was shown to activate the nucleotide-binding oligomerization domain-like receptor family pyrin domain-containing 3 (NLRP3) inflammasome in human monocytic THP-1 cells and mouse dendritic cells, resulting in IL-1β secretion (31). Whether the same inflammasome is activated in primary skin keratinocytes resulting in IL-1β production is unknown but warrants further investigation. Although minor differences existed in the cytokine secretion profiles between the epidermal models and primary keratinocytes, this probably relates to the structural differences between the two systems (e.g., monolayer versus multilayer).

Although not an *in vivo* system, the organotypic models used may prove useful in studying early steps in pathogenesis and intracellular signaling. In particular, this study found that T. equinum infection led to the upregulation of matrix metalloproteases (MMPs) and their regulators, tissue inhibitors of metalloproteases (TIMPs), as part of the response to remodel the extracellular matrix after damage induction. Increases in both gene expression and activity for MMPs and TIMPs in epithelial tissue/cells have been reported in response to C. albicans (32) and Cryptococcus neoformans (33) as well as other fungi (34). To our knowledge, this is the first description of the upregulation of either MMP or TIMP gene expression by skin epithelium in response to dermatophyte infection. In common with the aforementioned studies, we find upregulation of *timp1* as well as *mmp1*. Notably, the expression kinetics for these two genes differ, with *timp1* increasing slightly after *mmp1*, corresponding to its role as an inhibitor of MMP1 activity. The increase in *timp1* expression is particularly noteworthy, given that TIMP1-knockout mice show improved clearance of Pseudomonas aeruginosa from mucosal surfaces (cornea and respiratory surfaces) (35), suggesting that the MMP/TIMP complexes play a significant role in clearing microbial infections. As well as modulating MMP activity, TIMP1 also induces cytokine-like effects, influencing cell growth, differentiation, and apoptosis (36). Thus, MMP/TIMP complexes are potentially important both in clearing dermatophyte infection and in resolving the tissue/cellular damage resulting from these infections, by either remodeling the underlying extracellular matrix or by inducing cell growth and differentiation.

T. equinum infection also led to the upregulation of COX2, the enzyme responsible for the formation of prostaglandins (e.g., PGE2). Although little is known regarding the role of COX2 and PGE2 in fungal disease, they have been shown to have several immunological effects in host responses to fungi, including immunosuppression (37), inhibition of antifungal immunity (38), and modulating cytokine phenotypes in response to fungal infection (39). Likewise, increases in PGE2 production during fungal infections have also been noted (38, 40). Thus, the increased levels of COX2 may represent a resolution mechanism acting to “switch off” the antifungal responses once the fungus has been cleared.

One of the key virulence attributes of fungi, including dermatophytes, is the ability to change morphology from a yeast or conidium to a filamentous form or hyphae (5, 27, 41). Since conidia are the “spore” form of the dermatophytes, it would seem appropriate that the keratinocyte response is targeted against the invasive, growing hyphal form rather than the conidia of the dermatophytes. In the EpiDerm model, heat-killed conidia did not cause damage or cytokine release for any of the five dermatophytes tested, indicating that live fungi are required to initiate the immune response. We previously demonstrated that oral and vaginal epithelial cells were able to discriminate between the yeast and hyphal forms of C. albicans via a MAPK-based signaling mechanism that resulted in the activation of the c-Fos transcription factor and the MAPK regulator MKP1 (15, 16). C. albicans also activates the NF-κB pathway in epithelial cells to induce cytokines (15) and the PI3K pathway, which functions to prevent damage induced by the fungus (20). These pathways appear to work in unison to orchestrate an optimized proinflammatory effector response.

Notably, no data exist with regard to intracellular signaling pathways or transcription factors activated by dermatophytes in skin keratinocytes. Thus, our study is the first to characterize these responses, demonstrating that a similar effector response, comprising MAPK, MKP1, c-Fos, proinflammatory genes, and cytokines, is induced in skin keratinocytes during dermatophyte infections. Interestingly, T. equinum did not activate the PI3K pathway despite causing damage, suggesting that either this damage-associated signaling response is not activated by T. equinum or this response differs between epithelial cells and skin keratinocytes. If the latter, our signaling data would suggest that damage protection is probably mediated by the MAPK pathways, as these are the main pathways activated. Like the PI3K pathway, no obvious pattern to NF-κB activation was evident in response to dermatophyte infection. Of relevance, keratinocyte activation is often correlated with damage induction. However, our data indicate that keratinocyte activation is almost certainly the consequence of dermatophyte detection rather than cellular damage, since signaling (ERK1/2, c-Fos, MKP1, COX2) responses are detected very early (within minutes/hours postinfection), when no damage is induced. In addition, GM-CSF and IL-8 are released, which are generally regarded as non-damage-associated cytokines.

Despite our findings, there are a number of limitations to our model system. First, the limited availability of primary keratinocytes limited certain aspects of the study to the response of just one species. T. equinum was selected because it is a zoophilic dermatophyte that produces an intermediate infection. It did not immediately kill the EpiDerm tissues, suggesting that cells exposed to T. equinum would have time to produce a response. T. equinum also produces abundant conidia (14). Second, although unlikely to be typically encountered by keratinocytes *in vivo*, an MOI of 10 was used to ensure that all the keratinocytes were interacting with dermatophytes and that uniform and reproducible responses were elicited and detected. Irrespective of these concerns, evidence is now accumulating to suggest that MAPK-based innate immune responses observed in mucocutaneous tissues are highly conserved, not only between epithelial and keratinocyte cell types but also between hyphal forms of different fungi (this study) (15, 16, 18). As such, this identifies both fungal hyphae and host signaling pathways as potential targets for future antifungal therapy.
